# Exploring the Structural Diversity in Inhibitors of α-Synuclein Amyloidogenic Folding, Aggregation, and Neurotoxicity

**DOI:** 10.3389/fchem.2018.00181

**Published:** 2018-05-25

**Authors:** Sukanya Das, Tara L. Pukala, Scott D. Smid

**Affiliations:** ^1^Discipline of Pharmacology, Adelaide Medical School, Faculty of Health Sciences and Medicine, University of Adelaide, Adelaide, SA, Australia; ^2^Discipline of Chemistry, School of Physical Sciences, Faculty of Sciences, University of Adelaide, Adelaide, SA, Australia

**Keywords:** α-synuclein, amyloid inhibition, 2-D08, transilitin, honokiol, punicalagin, dibenzyl imidazolidine

## Abstract

Aggregation of α-Synuclein (αS) protein to amyloid fibrils is a neuropathological hallmark of Parkinson's disease (PD). Growing evidence suggests that extracellular αS aggregation plays a pivotal role in neurodegeneration found in PD in addition to the intracellular αS aggregates in Lewy bodies (LB). Here, we identified and compared a diverse set of molecules capable of mitigating protein aggregation and exogenous toxicity of αSA53T, a more aggregation-prone αS mutant found in familial PD. For the first time, we investigated the αS anti-amyloid activity of semi-synthetic flavonoid 2′, 3′, 4′ trihydroxyflavone or 2-D08, which was compared with natural flavones myricetin and transilitin, as well as such structurally diverse polyphenols as honokiol and punicalagin. Additionally, two novel synthetic compounds with a dibenzyl imidazolidine scaffold, Compound 1 and Compound 2, were also investigated as they exhibited favorable binding with αSA53T. All seven compounds inhibited αSA53T aggregation as demonstrated by Thioflavin T fluorescence assays, with modified fibril morphology observed by transmission electron microscopy. Ion mobility-mass spectrometry (IM-MS) was used to monitor the structural conversion of native αSA53T into amyloidogenic conformations and all seven compounds preserved the native unfolded conformations of αSA53T following 48 h incubation. The presence of each test compound in a 1:2 molar ratio was also shown to inhibit the neurotoxicity of preincubated αSA53T using phaeochromocytoma (PC12) cell viability assays. Among the seven tested compounds 2-D08, honokiol, and the synthetic Compound 2 demonstrated the highest inhibition of aggregation, coupled with neuroprotection from preincubated αSA53T *in vitro*. Molecular docking predicted that all compounds bound near the lysine-rich region of the N-terminus of αSA53T, where the flavonoids and honokiol predominantly interacted with Lys 23. Overall, these findings highlight that (i) restricted vicinal trihydroxylation in the flavone B-ring is more effective in stabilizing the native αS conformations, thus blocking amyloidogenic aggregation, than dihydroxylation aggregation in both A and B-ring, and (ii) honokiol, punicalagin, and the synthetic imidazolidine Compound 2 also inhibit αS amyloidogenic aggregation by stabilizing its native conformations. This diverse set of molecules acting on a singular pathological target with predicted binding to αSA53T in the folding-prone N-terminal region may contribute toward novel drug-design for PD.

## Introduction

Parkinson's disease (PD) neuropathology is characterized by loss of dopaminergic neurons, and in most cases, deposition of Lewy bodies in the brain (Hughes et al., 1992). Aggregation and amyloid formation of the cytosolic protein α-Synuclein (αS), in both intracellular and extracellular areas, have been implicated in the formation of Lewy pathology (LP) and degeneration of dopaminergic neurons in the substantia nigra pars compacta region of the brain (Baba et al., 1998; Volpicelli-Daley et al., 2011; Luk et al., 2012; Iyer et al., 2014). Human αS is a 140-amino acid residue polymorphic protein consisting of a membrane binding N-terminal region, a non-amyloid β component (NAC) region and an acidic C-terminal tail. This protein is associated with key biological activities such as vesicle trafficking, maintenance of the synaptic SNARE complex and vesicle pools, and regulation of dopamine (DA) metabolism (Cabin et al., 2002; Sidhu et al., 2004; Chandra et al., 2005; Cooper et al., 2006). Along with the sporadic form of PD caused by wild type αS, a missense point mutation in the *snca* gene results in the more aggregation prone mutant αSAla53Thr (αSA53T), which has been found to be associated with a familial form of PD (Polymeropoulos et al., 1997; Narhi et al., 1999; Li et al., 2001; Papadimitriou et al., 2016). Since current treatments for PD aim only at replacing DA loss, and provide only transient symptomatic relief, there is an urgent need for treatments that can directly modify disease progression. Therefore, inhibition of αS aggregation, or its pathological mutant αSA53T, provides a disease-modifying therapeutic approach for PD, inclusive of its familial form.

The amyloidogenic aggregation of an intrinsically disordered protein (IDP) such as αS involves formation of heterogeneous and transient assemblies early in its aggregation pathway, that act as a precursor for fibrillization (Bousset et al., 2013). Considering the difficulty in gaining structural information on these conformational assemblies, ion mobility-mass spectrometry (IM-MS) is employed to monitor soluble αS conformations during the early aggregation process. IM-MS is a sensitive tool for structural study of conformational folding and other characterization (Lanucara et al., 2014), and has been used for high-throughput screening of amyloid inhibitors (Young et al., 2014). It allows measurement of both the mass and collision cross section (CCS) of an ion, and thereby can provide information on the structural changes of amyloidogenic proteins such as αS during aggregation and binding of small molecules (Bernstein et al., 2004; Vlad et al., 2011; Liu et al., 2015). Combined with kinetic analysis of fibrillization (such as from thioflavin T fluorescence assays) and visualization of the fibrils using transmission electron microscopy (TEM), IM-MS provides insights on the effect of exogenous compounds on native αSA53T conformations *in vitro*. This can inform on how such compounds interact during the early stage of toxic amyloidogenic aggregation. Evaluation of αSA53T neurotoxicity also provides valuable information on the toxic nature of the observed aggregation products, and possible neuroprotective effects of small molecule aggregation inhibitors.

Various natural compounds have been shown to inhibit αS aggregation or participate in remodeling of its fibrillization pathway (Zhu et al., 2004; Masuda et al., 2006; Bieschke et al., 2010; Morshedi et al., 2015). The polyphenolic flavonoids baicalein and epigallo-catechin-3-gallate (EGCG) are amongst these (Hong et al., 2008; Bieschke et al., 2010). Structurally, baicalein possesses vicinal trihydroxyl groups only in the flavone A-ring, whereas EGCG possesses vicinal trihydroxylation in the B-ring and in the gallic acid moiety. Gallic acid, by itself, also demonstrated inhibition of αSA53T aggregation (Liu et al., 2014). A comprehensive study on flavonoid-induced inhibition of αS aggregation has shown that vicinal dihydroxylation or trihydroxylation improves inhibition irrespective of ring position (Meng et al., 2009). However, except for baicalin, other reported flavonoid inhibitors lack the localized single ring vicinal di or tri hydroxyl groups. Thus, the importance of position and extent of hydroxylation of flavonoids involved in inhibiting αS aggregation and neurotoxicity is unclear. In this study, we have investigated the anti-amyloid effect of semi-synthetic, 2′, 3′, 4′ trihydroxy flavone (2-D08) that has only vicinal trihydroxylation in the B-ring. This flavone has shown improved inhibition of amyloid β (Aβ) aggregation and toxicity through its localized B-ring trihydroxylation (Marsh et al., 2017). We compared it with myricetin, a known inhibitor of αS aggregation (Meng et al., 2010; Liu et al., 2015) which also has vicinal trihydroxylation in the B-ring along with -OH groups at flavone 3, 5, and 7 positions. Transilitin was also investigated here to compare the effect of dihydroxylation in both A and B rings. Our study provides information on the role of select B-ring trihydroxylation for modulation of αS aggregation and toxicity.

**Figure 1:**
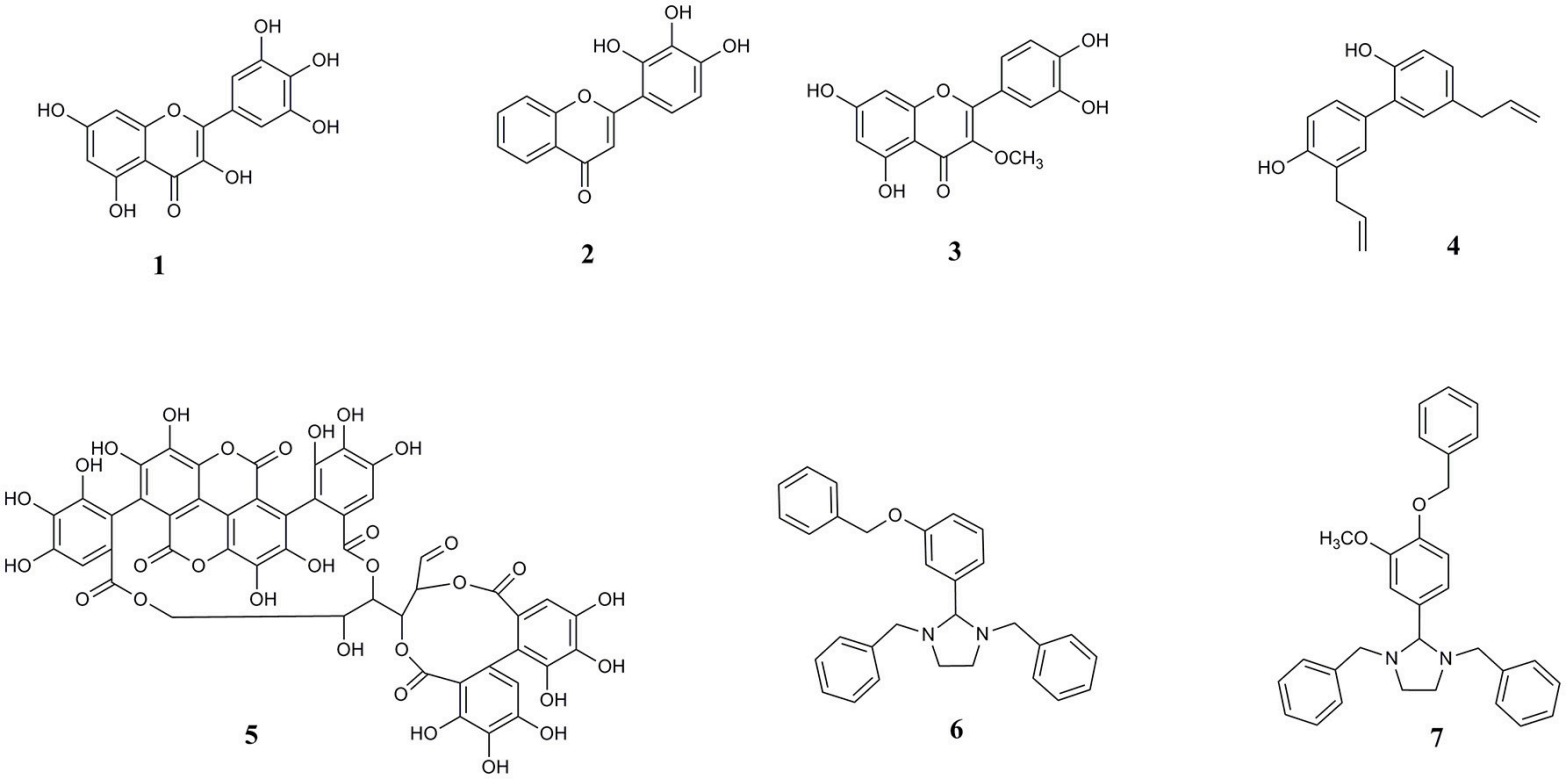
Chemical structures of **(1)** myricetin, **(2)** 2′, 3′, 4′ trihydroxy flavone or 2-D08, **(3)** transilitin, **(4)** honokiol, **(5)** punicalagin, **(6)** 1,3-dibenzyl-2-[3-(benzyloxy) phenyl] imidazolidine or Compound 1, and **(7)** 1,3-dibenzyl-2-[4-(benzyloxy)-3-methoxyphenyl] imidazolidine or Compound 2.

Other bioactive polyphenols investigated here for the first time are the natural lignan compound honokiol and ellagitannin punicalagin. Honokiol has demonstrated anti-amyloid effects against amyloid beta (Aβ) (Hoi et al., 2010; Das et al., 2016). Punicalagin, found in pomegranate, is also a known inhibitor of Aβ protein aggregation and has antioxidant and anti-inflammatory properties (Yaidikar et al., 2014; Yaidikar and Thakur, 2015; Das et al., 2016). Extending the structural diversity of molecules that can act on a common target of pathological αS aggregation, we also included two synthetic imidazolidine compounds, termed Compound 1 (1,3-dibenzyl-2-[3-(benzyloxy) phenyl] imidazolidine) and Compound 2 (1,3-dibenzyl-2-[4-(benzyloxy)-3-methoxyphenyl] imidazolidine), both bearing a five-membered heterocyclic (FMH) imidazolidine ring scaffold. We previously identified these compounds through virtual screening of the ZINC chemical database and highlighted the importance of this molecular scaffold with regards to preventing amyloid β aggregation (Das and Smid, 2017). A summary of the compounds investigated here is provided in Figure 1.

## Materials and methods

### Reagents and chemicals

Myricetin, 2-D08, honokiol, and punicalagin were obtained from Sigma-Aldrich (Castle Hill, VIC, Australia). Transilitin was kindly provided by Dr. Peter Duggan at CSIRO Materials Division (Clayton South, VIC, Australia). Synthetic imidazolidine Compound 1 and Compound 2 were sourced from Specs (The Netherlands) and ChemDiv (U.S.A.) respectively, both screening compound manufacturers. Purity of Compound 1 was assessed to be >97% through ^1^H NMR analysis (Supplementary Figure 1), Compound 2 was certified with a purity of >97%.

Thioflavin T, thiazolyl blue tetrazolium bromide (MTT), trypan blue, DMSO, Roswell Park Memorial Institute 1640 (RPMI) medium and fetal calf serum (FCS) were obtained from Sigma-Aldrich (St Louis, MO, USA). Non-essential amino acids (NEAA), penicillin/streptomycin, 1 × trypsin EDTA, and phosphate buffered saline (PBS) at pH 7.4 were obtained from Thermo Fisher Scientific, (Scoresby, VIC, Australia). Bovine serum albumin (BSA) was obtained from Bovogen Biologicals (East Keilor, VIC, Australia). Ammonium acetate was obtained from Sigma-Aldrich (Castle Hill, VIC Australia).

### Protein expression and purification

αSA53T was expressed and purified as previously described (Volles and Lansbury, 2007). Cells were grown in normal lennox broth (LB) medium. Protein was purified by size exclusion chromatography using a Superdex 200 SEC column (Bio Rad), with a flow rate of 0.5 ml/min, in 20 mM ammonium acetate buffer. The purity of αSA53T was confirmed by mass spectrometry and samples were lyophilized and stored at −80°C until required.

### Preincubation of αSA53T and compound preparation

αSA53T was dissolved in 50 mM ammonium acetate buffer (pH not adjusted) to make 1 mM stock solution, dispensed into aliquots and immediately frozen at −80°C until required. All test compounds were initially diluted in DMSO and then in 50 mM ammonium acetate to their final stock concentrations prior to incubation with αSA53T. The final concentration of DMSO in each experiment was < 1%. For the cell viability assay, 50 μM αSA53T alone or in the presence of each compound (100 μM) in 50 mM ammonium acetate buffer was shaken at 300 rpm for 72 h at 37°C.

### Thioflavin T fluorescence assay

Thioflavin T (ThT) binds to β sheet rich structures present in amyloid fibrils, with fluorescence increasing proportionally to the quantity of fibrils present in solution. ThT (final concentration 100 μM) was added to wells in a Greiner 96-well plate together with αSA53T (100 μM), in the absence or presence of each test compound (either at 200 or 100 μM) in 50 mM ammonium acetate buffer to a total volume of 100 μl in each well. Fluorescence was measured at 37°C every 30 min for 100 h using a Fluostar Optima plate reader (BMG Lab technologies, Australia) with a 440/490 nm excitation/emission filter. The ThT assay was performed in duplicate and repeated three times. Results were normalized to blank values (ThT alone in 50 mM ammonium acetate buffer).

### Transmission electron microscopy (TEM) imaging

TEM was used to visualize αSA53T aggregates and fibrils and investigate the effects of selected compounds on fibril morphology. Samples were prepared by adding 5–10 μl of protein solution taken directly from the ThT assay after 100 h to a 400 mesh formvar carbon-coated nickel electron microscopy grid (Proscitech, Kirwan, QLD, Australia). After 1 min, this sample was blotted using filter paper. Ten microliters of contrast dye containing 2% uranyl acetate was then placed on the grid, left for 1 min and blotted with filter paper. Grids were then loaded onto a specimen holder for analysis using a FEI Tecnai G2 Spirit Transmission Electron Microscope (FEI, Milton, QLD, Australia). Sample grids were viewed using a magnification of 34,000–92,000X. Grids were extensively scanned manually in search of fibrils and representative images were taken.

### Ion mobility-mass spectrometry (IM-MS)

Fifty micromolar αSA53T was prepared for IM-MS experiments in 50 mM ammonium acetate buffer in the absence and presence of each compound at a molar ratio of 1:2 (protein: compound). Samples were allowed to fibrillize by incubation at 37°C with constant shaking at 300 rpm. IM-MS analysis was performed on an Agilent 6560 Ion Mobility Q-ToF spectrometer with samples introduced by nanoelectrospray ionization through platinum-coated capillaries (made in-house). Ions were analyzed in the positive mode, with parameters systematically selected to achieve optimal signal while avoiding any analysis induced structural transitions (full details of optimization will be reported in a manuscript currently in preparation). Typical instrument parameters included; capillary voltage 1,700 V, fragmentor voltage 400 V, gas temperature 0°C, gas flow 2 l/min, trap fill time 20000.0 μs, trap release time 4000.0 μs and CCS measurement was made using a multifield approach varying the IM drift tube voltage between 1,200 and 1,700 V. The acquired spectra were processed using Qualitative Analysis B.07.00 and IM-MS browser B.07.01 (both Agilent, Santa Clara, USA).

### Neuronal cell culture, treatment, and viability measurements

Rat phaeochromocytoma cells (Ordway PC12) displaying a semi differentiated phenotype with neuronal projections were kindly donated by Professor Jacqueline Phillips (Macquarie University, NSW, Australia) [21]. Cells were maintained in Roswell Park Memorial Institute 1640 (RPMI) media with 10% fetal calf serum (FCS), 1% L-glutamine, 1% non-essential amino acids, and 1% penicillin/streptomycin. Cells were seeded at 2 × 10^4^ cells per well in RPMI with 10% FCS. PC12 cells were equilibrated for 24 h before treatment with preincubated αSA53T or preincubated αSA53T in presence of each test compound. Cells were then incubated for 48 h at 37°C, 5% CO_2_ prior to measurement of cell viability. After 48 h, PC12 cell viability was determined using the thiazolyl blue tetrazolium bromide (MTT) assay. After incubation, 96-well plates had all media removed and replaced with serum-free media containing 0.25 mg/ml of MTT. The plate was further incubated for 2 h at 37°C with 5% CO_2_, then the MTT solution was removed and cells were lysed with DMSO. Absorbance was measured at 570 nm using a Synergy MX microplate reader (Bio-Tek, Bedfordshire, UK).

### Statistical analysis

Area under the curve analysis for Thioflavin T (ThT) fluorescence data was interrogated using a one-way analysis of variance (ANOVA) with a Dunnett's multiple comparisons test to determine the significance of each tested compound's effect vs. αSA53T alone. Data obtained from the MTT assay was analyzed via a one-way ANOVA to assess neuronal cell viability with a Holm–Sidak's multiple comparison test used to determine the significance level for each test compound interacting with αSA53T. A significance value of *p* < 0.05 was used for all experiments. Data analysis and production of graphs was performed in GraphPad Prism 6 for Windows (GraphPad Software, San Diego, USA).

### Molecular docking of compound optimized structure binding to α synuclein

To gain insight into the binding interactions of each selected compound (ligand) with αSA53T, all ligand structures were first optimized and then individually docked to αSA53T with 300 iterations per docking. Previous experimental evidence suggested that the flavonoid binding site in α synuclein is near the lysine- rich region of the N-terminus, with Lys21 or Tyr39 playing a pivotal role (Meng et al., 2009). Therefore, the docking search space was created centering this binding site with a radius of 25 Å covering these N-terminus residues. Ligand equilibrium molecular geometries were optimized using a density functional theory (DFT) method that utilizes the Becke-Lee-Yang-Parr three-parameter hybrid functional (B3LYP) to ascertain accurate bond distances, angles, dihedrals and optimized conformations in the lowest energy state, using the Gaussian09 package of codes (Frisch et al., 2016). A large basis set, aug-cc-pVDZ, was used in approximation of optimized geometry for each ligand. DFT-B3LYP level of calculations combined with a larger basis set is considered as a standard and reliable method for estimating optimized molecular geometry (El-Azhary and Suter, 1996). Fully optimized ligand structures were then allowed to dock with the model αSA53T. Since the full sequence αSA53T structure was not available, the solid-state NMR of pathogenic α synuclein fibril (PDB ID: 2N0A) was obtained from protein data bank and residue 53 mutated from alanine to threonine to generate the model protein monomer of αSA53T (Tuttle et al., 2016). The resultant αSA53T protein conformation, without any further adjustment, was used for docking, which employed CLC drug discovery workbench, version 2.4.1 using a PLANTSplp empirical scoring function (Korb et al., 2009).

## Results

### Significant inhibition of αSA53T amyloid fibril formation by all test compounds

**Figure 2:**
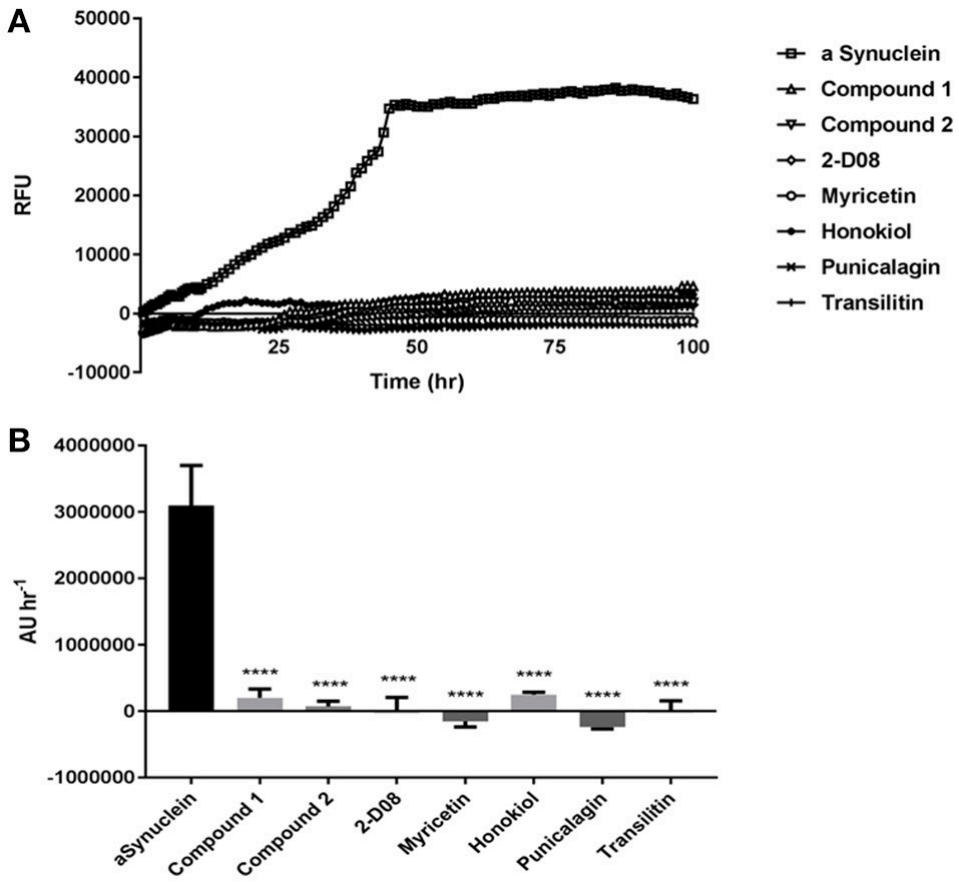
**(A)** ThT fluorescence assay representing kinetics of (100 μM) αSA53T fibrillisation over 100 h, alone and in the presence of each test compound at a 1:2 (protein: compound) molar ratio. **(B)** Representative area under the curve (AUC) measurements demonstrating significant reductions in ThT fluorescence for each test compound at a 1:2 (protein: compound) molar ratio. [****p < 0.0001, *F*_(7, 17)_ = 15.96; mean ± SEM of *n* = 3 experiments].

The amyloid fibrillization of αSA53T was measured by a ThT-based kinetic assay, which demonstrated a gradual increase in fluorescence (relative fluorescence unit or RFU) up to the first 50 h and then stabilized over the remainder of the assay, indicating αSA53T underwent a conformational transition to a cross-β-sheet rich structure characteristic of amyloid fibrils and aggregates (Figure 2A). All test compounds: 2-D08, myricetin, transilitin, honokiol, punicalagin, Compound 1 and Compound 2 inhibited the ThT fluorescence over the entire 100 h time course of the assay at a 1:2 (protein: compound) molar ratio. Area under the curve analysis showed extensive and significant overall inhibition of fibril formation in the presence of each of the test compounds at a 1:2 (protein: compound) molar ratio (Figure 2B). Further ThT assays at a 1:1 (protein: compound) molar ratio also exhibited significant fibril inhibition, though Compound 1 was the least effective among all compounds tested (Supplementary Figures 2a,b). The mean RFU along with the respective standard error of mean (SEM) values for each compound are provided as Supplementary Table 1.

### Transmission electron microscopy of αSA53T fibrils and aggregates

**Figure 3:**
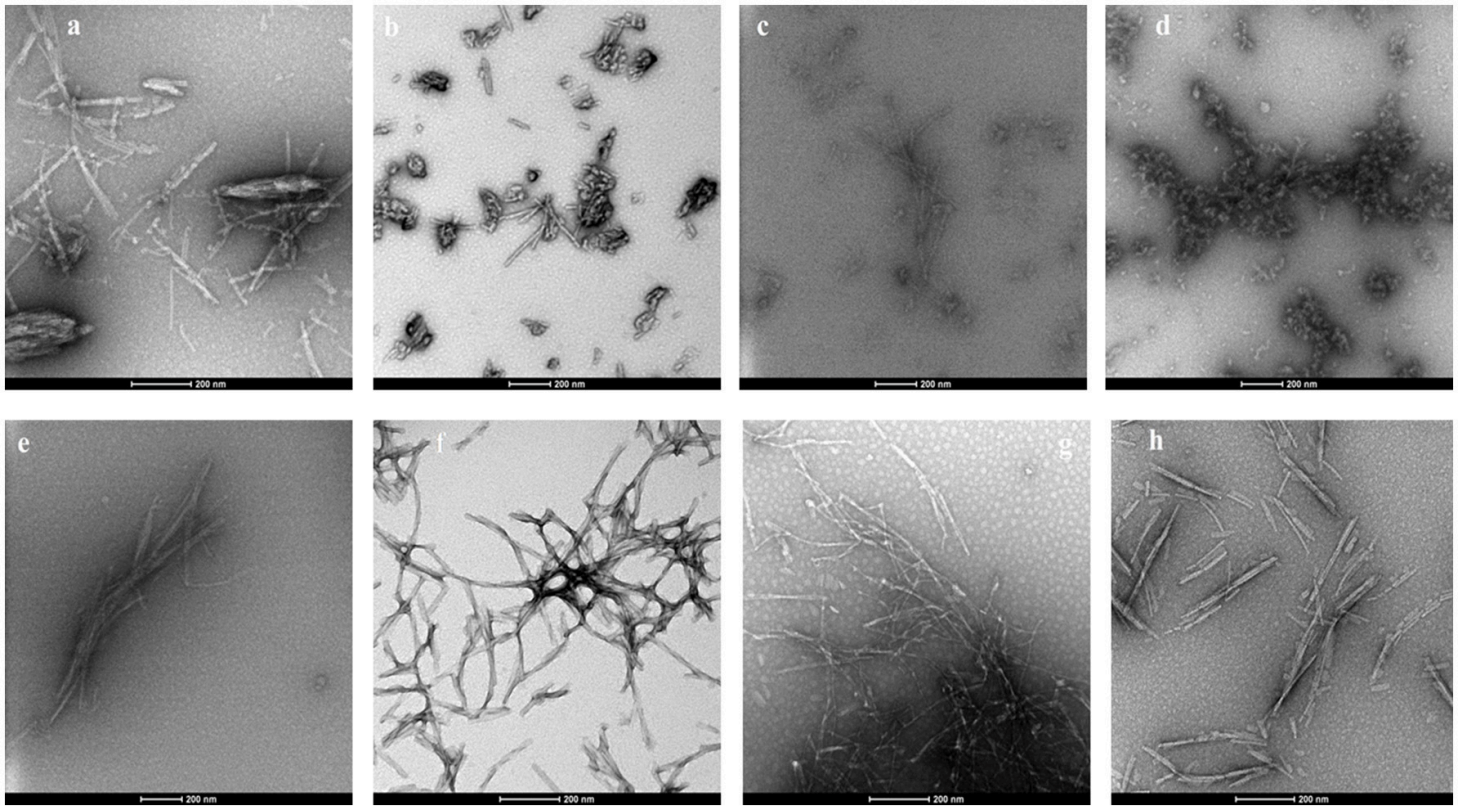
Representative transmission electron micrographs of αSA53T fibril and aggregate formation, following 100 h incubation alone and with each test compound at a 1:2 (protein: compound) molar ratio; **(a)** αSA53T alone and in presence of **(b)** Compound 1, **(c)** Compound 2, **(d)** 2-D08, **(e)** myricetin, **(f)** honokiol, **(g)** punicalagin, and **(h)** transilitin. Scale bar: 200 nm.

False positives for ThT inhibition can occur when some polyphenols undergo spontaneous oxidation in aqueous solution and strongly quench ThT fluorescence, and this also might have resulted in negative RFU for myricetin and punicalagin (Coelho-Cerqueira et al., 2014). Therefore, the inhibition of fibrillization observed in ThT assays was further confirmed by TEM, an essential qualitative technique to characterize the morphology of amyloid fibril and aggregate formation. The morphology of αSA53T fibrils and aggregates appeared to be affected by incubation with each of the test compounds at a 1:2 (protein: compound) molar ratio, after 100 h incubation (Figures 3a–h). TEM evidence demonstrated that αSA53T alone formed dense fibrillar aggregates where several fibrils were intertwined and arranged as rope-like mature amyloid fibrils, similar to those observed previously (Bharathi et al., 2007; Figure 3a). Incubation with Compound 2, myricetin and transilitin resulted in shorter, thinner, and loosely attached fibrils, but in low abundance (Figures 3c,e,h). Incubation with honokiol produced loosely attached, short and long fibrils while punicalagin incubation produces loosely attached, thin fibrils that were comparatively longer (Figures 3f,g). Incubation with 2-D08 resulted in amorphous aggregates of very short fibrils (Figure 3d), while incubation with Compound 1 produce short fibrils and small dense aggregates (Figure 3b). In the presence of all test compounds, no rope-like aggregates were observed compared to αSA53T alone. Additional TEM images following 50 h incubation highlighted the formation of pre-fibrillar structures by αSA53T alone. In the presence of all compounds except punicalagin, small aggregate-like structures were observed. Punicalagin incubation resulted in a few protofibrillar αSA53T structures similar to those that were observed following 100 h incubation (Supplementary Figure 3).

### Preservation of early αSA53T conformations by test compounds monitored using IM-MS

**Figure 4:**
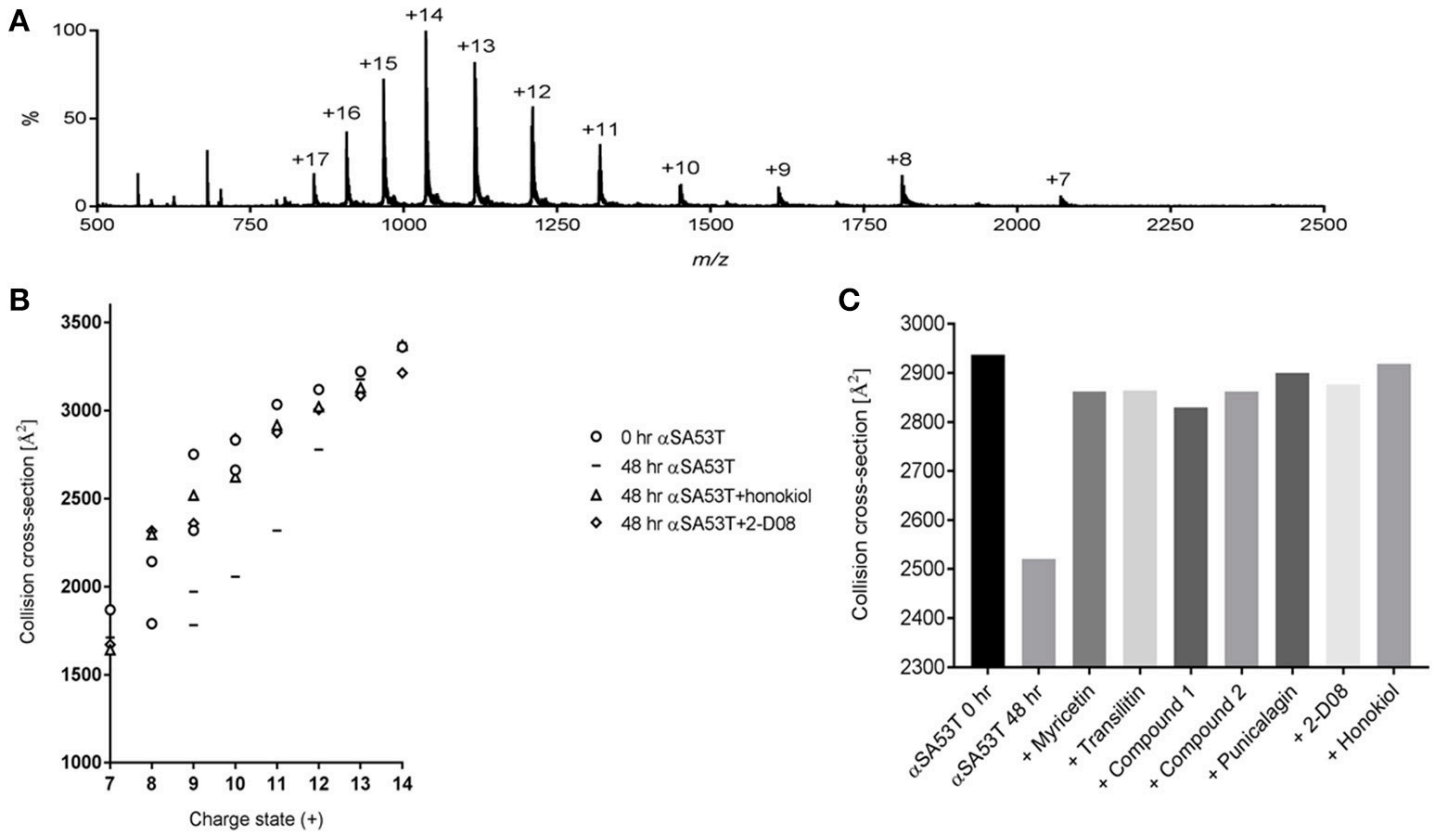
**(A)** IM-MS spectrum of αSA53T (50 μM) in 50 mM ammonium acetate. **(B)** Plot of CCS vs. charge state for the dominant peaks in the IM-MS spectrum measured for αSA53T at 0 h (circles) and following incubation in the absence (squares) and presence of 1:2 (protein: compound) honokiol (triangles) or 2-D08 (diamonds). Where multiple features were observed in the ATD, CCS is plotted for each feature. **(C)** Experimentally derived CCSs for the +11-charge state (*m/z* 1,317) of αSA53T following 48 h incubation at 37°C with shaking at 300 rpm.

IM-MS was utilized to investigate the αSA53T conformational changes occurring early during aggregation in the presence and absence of each test compound. An effective amyloid inhibitor compound would not only prevent fibril formation, but also preserve the protein in its natively unfolded state. IM-MS has important application for investigating the early aggregation phenomena of amyloidogenic proteins, since the unfolded native proteins convert into a compact folded conformation that precedes fibrillization (Smith et al., 2010; Liu et al., 2014). The mass spectrum of αSA53T prior to incubation displayed a broad charge state distribution consistent with a natively unstructured protein (Konermann and Douglas, 1998; Figure 4A). The measured CCSs for monomeric αSA53T ions prior to incubation were plotted in Figure 4B, and are in agreement with previously reported IM-MS measurements of the αSA53T monomer (Liu et al., 2011). Lower charge states having a lower CCS represent the population of ions with more compact structure, while from charge states +8 to +9 the CCS measured increases significantly, indicative of a transition from compact to extended structures. A similar observation was reported previously for wild type α-synuclein (Bernstein et al., 2004).

Following 48 h incubation, IM-MS analysis showed that αSA53T ions of both lower and higher charge states from +7 to +12 underwent a structural collapse as indicated by a notable decrease in measured CCSs. Notably, when αSA53T was incubated in the presence of inhibitor compounds, the measured CCS values of the monomers revealed that the protein mostly remained in its native conformation after 48 h, with honokiol or 2-D08 shown to be most effective (Figure 4B). Among the flavones, CCSs of αSA53T in the presence of myricetin or 2-D08 or transilitin were comparable.

To simplify the analysis of αSA53T conformations in the presence of each test compound, we selected a single charge state, +11, for comparison of CCSs based on the data shown in Figure 4B. This charge state was selected as ion populations at higher charge states tend to display a single, narrow feature in the arrival time distribution and it showed remarkable collapse of unfolded conformations, from 2,937 to 2,520 Å^2^ following 48 h of incubation. Incubation of αSA53T in the presence of punicalagin, myricetin, transilitin, Compound 2 and Compound 1 preserved the CCS of the 11+ ions close to the preincubation measurement after 48 h (Figure 4C). Detailed CCSs measurements of all these compounds for each charge state is provided in Supplementary Figures 4a–e. Overall, all seven test compounds demonstrated inhibition of the early structural collapse of αSA53T during aggregation, with honokiol and 2-D08 being the most effective as evidenced by preventing the decrease in CCSs.

### Effects of compounds on fibrilar αSA53T mediated neuronal toxicity

**Figure 5:**
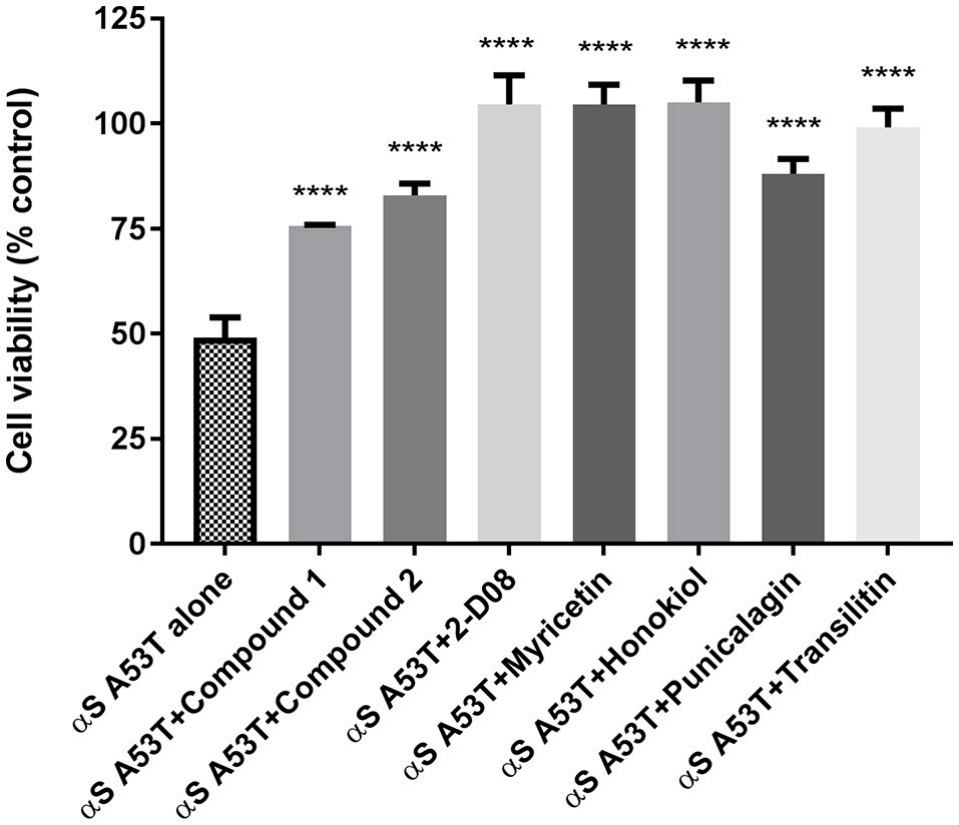
Cell viability of PC12 cells described by an MTT assay following 48 h incubation of preincubated (for 72 h) 50 μM αSA53T alone or with each test compounds at a 1:2 (protein: compound) molar ratio. [^****^p < 0.0001; *F*_(3, 11)_ = 21.61 for Compound 1, Compound 2, and punicalagin and ^****^p < 0.0001; *F*_(4, 17)_ = 21.77 for 2-D08, myricetin, honokiol, and transilitin; mean ± SEM of *n* = 4 experiments].

Analysis of cell viability as determined by the MTT assay demonstrated that preincubated αSA53T (50 μM) alone evoked about 50% loss of cell viability over 48 h (Figure 5). In contrast, preincubated αSA53T in the presence of each test compound at a 1:2 (protein: compound) molar ratio prevented the loss of cell viability significantly (Figure 5). PC12 cells exposed to preincubated αSA53T in the presence of 2-D08, myricetin, or honokiol (100 μM each) did not show any loss of cell viability. This finding implies that interaction of these compounds with αSA53T inhibited the neurotoxicity of the resultant fibrils toward PC12 cells *in vitro*. The two synthetic imidazolidines, Compound 1 and Compound 2 also significantly mitigated the loss of cell viability compared to αSA53T alone, however, the degree of neuroprotection was not as pronounced as the other natural test compounds (Figure 5). In a 1:1 (protein: compound) molar ratio, only Compound 2, 2-D08, myricetin, honokiol, and punicalagin-treated significantly reversed the loss of cell viability from αSA53T (Supplementary Figure 5). The mean percentage cell viability along with respective SEM values for each compound are provided as Supplementary Table 2.

### Molecular modeling of optimized conformations of test compounds to αs A53T

**Figure 6:**
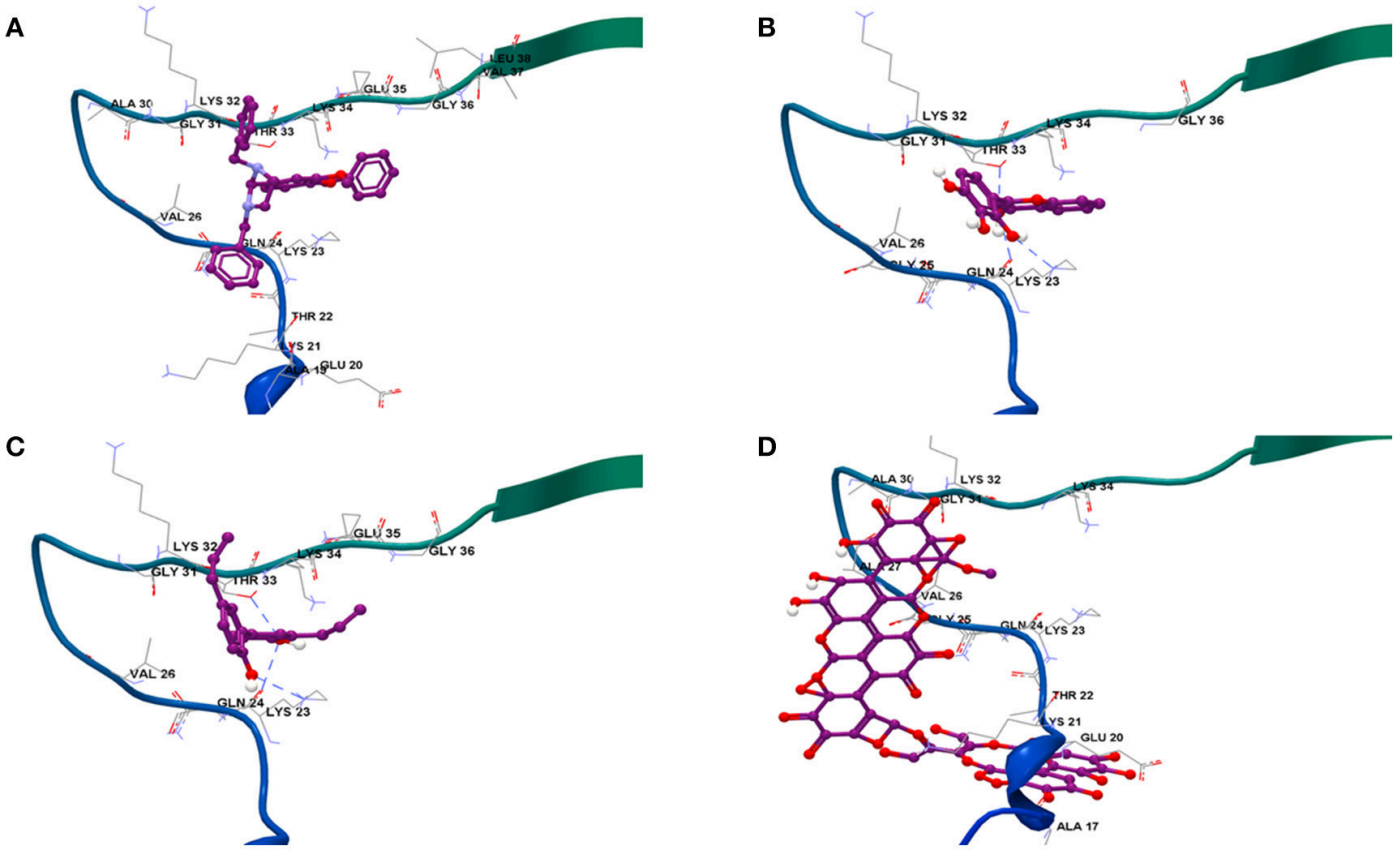
Binding modes of the four strongest inhibitor optimized ligand structures to αSA53T monomer: **(A)** Compound 2, **(B)** 2-D08, **(C)** honokiol, and **(D)** punicalagin.

Results from docking studies indicated that all the test compounds bound near the lysine-rich hinge area ranging from residue Ala 17 to Gly 36 in the predefined docking search space (Figures 6A–D and Supplementary Figures 6a–c). Both synthetic imidazolidines Compound 1 and Compound 2 displayed higher docking scores and steric interactions than the rest of the ligands; however, neither formed any H-bonding interactions (Table 1; Figures 6A,B). The two trihydroxy flavones, 2-D08 and myricetin, yielded similar scores, with myricetin forming more H-bonding interactions than 2-D08. Transilitin, a dihydroxy flavone, had similar docking and H-bonding scores as myricetin. The lignan honokiol had the highest score among the natural compounds, indicating strong interaction and H-bonding to Lys 23 and Thr 33 while the ellagitannin punicalagin had the lowest score, implying punicalagin might be a poor binder to αSA53T. H-bonding with key Lys residues, especially Lys 23 and Lys 21 was found in the case of all tested natural ligands.

**Table 1:** Docking profiles of seven test compounds to αSA53T monomer.

**Ligand**	**Docking score**	**H-bond forming residues**	**H-bond score**	**Steric interaction score**	**RMSD (Å)**
Compound 1	−69.23	−	0.00	−73.48	133.13
Compound 2	−65.53	−	0.00	−68.25	136.99
2-D08	−44.89	Lys 23, Thr 33	−6.00	−40.88	137.15
Myricetin	−41.82	Ala 19, Lys 21, 23, Thr 22, Gln 24, Gly 25	−10.45	−32.68	129.40
Honokiol	−58.36	Lys 23, Thr 33	−6.00	−55.55	137.42
Punicalagin	−38.00	Lys 21, Lys 23	−5.83	−38.28	131.27
Transilitin	−50.09	Lys 23, Lys 32, Thr 33, Lys 34	−10.00	−44.82	136.70

## Discussion

A growing number of studies have emphasized that accumulation or aggregation of α-synuclein plays a key role in neurodegeneration via mitochondrial and lysosomal dysfunction (Hashimoto et al., 2003; Mazzulli et al., 2016). A search for αS aggregation inhibitors possessing different molecular scaffolds, other than the commonly used dopamine analogs would be insightful for rational drug design. This study has identified and compared the neuroprotective properties of common and novel flavones as well as other polyphenols, along with two synthetic imidazolidine compounds, which can attenuate the toxic aggregation of αSA53T *in vitro*. The seven compounds studied here can be broadly divided into four categories, based on their structures; (i) the flavones: myricetin, 2-D08 and transilitin, (ii) neolignan: honokiol, (iii) ellagitannin: punicalagin, and (iv) synthetic dibenzyl phenyl imidazolidines: Compound 1 and Compound 2. Molecular docking provided additional insight into their probable binding sites and the nature of interactions that might contribute to structural modification of αSA53T during fibrillization, to result in the formation of non-toxic aggregates.

### Interaction of flavones and their effect on αSA53T conformation, fibrillization, and neurotoxicity

The three flavones studied here possessed a catechol-type vicinal tri-hydroxylation in the B-ring (myricetin and 2-D08) and di-hydroxylation in both A and B-rings (transilitin). A major aim of this study was to investigate how restricted tri-hydroxylation in the flavone B-ring (2-D08) impacts αS amyloid aggregation. TEM imaging revealed that 2-D08 altered the fibril morphology more significantly than myricetin or transilitin, despite similar ThT fluorescence outputs, in particular, TEM images after 50 h incubation revealed that 2-D08 modified the αSA53T active fibrillisation phase differently than myricetin or transilitin. The amorphous αSA53T aggregates arising from incubation with 2-D08 are likely to possess a reduced hydrophobic surface that is critical for ThT micellar binding, thus lowering fluorescence (Khurana et al., 2005). These amorphous aggregates resemble α-Synuclein treated with baicalein as observed in a previous study (Hu et al., 2016). Notably, 2-D08 showed an exclusive interaction with Lys 23, followed by Thr 33 and unlike transilitin and myricetin. Therefore, restriction of the *O*-quinone forming three -OH side groups in either the A or B-ring of the flavone might be favorable for binding to inhibit αS fibrillization as previously observed with baicalein (Hong et al., 2008). Nevertheless, the primary interaction of 2-D08 with αSA53T could be a low affinity, transient interaction between the protein and ligand rather than covalent modification of the Lys and Thr residues in the binding region, as we did not observe significant adduct formation in our IMMS study. Gallic acid had been shown to inhibit αSA53T structural collapse in a similar manner (Liu et al., 2014).

We previously reported that 2-D08 exerted a novel anti-aggregatory and neuroprotective effect against Aβ compared to transilitin and quercetin, which have A and C-ring hydroxylation (Marsh et al., 2017). In the present study, 2-D08 induced inhibition of αS aggregation and neuroprotection was as pronounced as myricetin, a generic amyloid aggregation inhibitor, and supports the idea that restricted B-ring tri-hydroxylation is adequate for targeting both αS and Aβ (Ono and Yamada, 2006; Hirohata et al., 2007; Zelus et al., 2012; Liu et al., 2015). From the TEM imaging, it appeared that transilitin modified the αSA53T fibrils in a similar way to myricetin, where only loose fibrils but no amorphous aggregates were seen. It is likely that these two flavones with A-ring hydroxylation share a common mechanism for inhibiting aggregation by reducing toxic amyloidogenic fibrils, whereas 2-D08 may inhibit amyloid fibrillization through formation of non-toxic amorphous aggregates. Consequently, within the flavone molecular scaffold, both 2-D08 and transilitin appear to be generic inhibitors of αSA53T and Aβ aggregation, similar to myricetin. Comparing the strong inhibitory effect of transilitin with the flavonoid G-500 suggests that additional hydroxyl groups other than the two pairs in the A and C-rings might be redundant (Meng et al., 2010). Together, our findings emphasize the previously reported idea that vicinal trihydroxylation in the flavone is favorable for strong inhibition of αS aggregation, coupled with its precise position in the B-ring (Meng et al., 2010).

IM-MS is an emerging analytical method that retains weak non-covalent interactions in the gas-phase and maintains protein conformations from solution phase (Konermann and Douglas, 1998; Sarni-Manchado and Cheynier, 2002; Wyttenbach and Bowers, 2011; Abzalimov et al., 2012). The IM-MS study presented here reinforces that soluble αSA53T undergoes a structural transition from natively unfolded to more compact amyloidogenic conformations at an early stage of aggregation, as measured by changes in CCSs. This is consistent with our ThT results, where the increasing fluorescence reaches its maximum by ~50 h, indicating the fibril plateau phase is reached in this time frame. Stabilization of unfolded αSA53T conformations to prevent amyloid formation was observed by IM-MS in the presence of all three flavones tested and was further supported by inhibition of ThT fluorescence during this early aggregation period.

No loss in PC12 cell viability was observed *in vitro* by preincubated αSA53T in the presence of 2-D08, even at an equimolar concentration, corroborating that the loose amorphous aggregates formed under these conditions are not amyloidogenic or toxic. The prevention of structural collapse by 2-D08 effectively inhibited the toxic amyloid formation of αSA53T, providing a degree of neuroprotection as pronounced as myricetin. Transilitin, on the other hand, was less effective in halting αSA53T amyloidogenic aggregation and neurotoxicity at an equimolar concentration. Since there is some degree of αSA53T folding as measured by CCSs in the presence of transilitin, this might account for the reduced neuroprotection at a lower concentration. Combining the anti-aggregation and neuroprotection results, we can infer that flavone tri-hydroxylation is more effective against toxic αSA53T amyloidogenic aggregation than di-hydroxylation. An understanding of the precise flavone structural requirements for strong inhibition of αS toxic aggregation would facilitate improved drug design. As exemplified with quercetin, hydroxylation in the flavone 3 and 7 positions are susceptible to thiol formation with cellular proteins rather than being neutralized in the cellular antioxidant network (Jacobs et al., 2011). Therefore, not having any hydroxylation in these positions while retaining a strong inhibitory effect seems a valuable strategy for improved drug design. Consequently, 2-D08 would be more favorable than myricetin to serve as a template for flavone-based inhibitors of αS toxic aggregation.

### Lignan interaction with αSA53T during fibrillization and neurotoxicity

Outside the flavonoid class of compounds, other small molecule polyphenolics such as lignans have recently gained attention for amyloid inhibition. In the present study, honokiol showed a profound effect on αSA53T aggregation, as indicated by the TEM imaging and ThT assay. TEM images of honokiol-treated αSA53T fibrils to some extent resembled analogous samples with punicalagin when comparing the long fibrils, and transilitin when comparing the short fibrils. These modified fibrils might account for the residual ThT fluorescence. As a strong inhibitor, honokiol prevented the structural collapse of native monomeric αSA53T at a very early stage of aggregation, as observed for 2-D08, although treatment with honokiol did not produce any amorphous aggregates. TEM images following 50 h incubation suggested that honokiol effectively inhibited αSA53T fibrillization at an early stage, in agreement with the ThT and IM-MS results. The demonstrated neuroprotection by preincubated αSA53T fibrils in the presence of honokiol highlights the non-toxic and non-amyloidogenic nature of the detached fibrils observed. Molecular docking results indicated that honokiol has the highest overall and steric binding scores among the natural compounds, while interacting strongly with key residues Lys 23 and Thr 33 through hydrogen-bonding, a similar interaction observed in the docking profile of 2-D08. This indicated a more sterically favorable interaction with this binding region to stabilize the unfolded αSA53T monomer. Nonetheless, honokiol lacks any *O*-quinone forming vicinal dihydroxyl structure like 2-D08 or other catechol-type flavones. Therefore, it might not covalently modify the target but interact through π-π stacking. Presumably, honokiol and tri-hydroxyflavones such as 2-D08 or myricetin might have different modes of interacting with αSA53T, nevertheless, they all result in similar degree of inhibition of amyloidogenic aggregation. Previously, honokiol was reported to be effective against toxicity related to Aβ and calcitonin aggregation (Guo et al., 2015; Das et al., 2016), and shown to be as effective as resveratrol and EGCG in an *in vivo* model of Aβ toxicity, inhibition of cholinesterase and metal chelation (Kantham et al., 2017). Together, honokiol has as pronounced an effect as the two tri-hydroxyflavones on inhibiting αSA53T aggregation and toxic amyloidogenic formation. Nonetheless, the molecular mechanism underlying this lignan based inhibition of αS aggregation is yet to be fully understood.

### Effect of ellagitannin on αSA53T fibrillization and neurotoxicity

Punicalagin, being the largest molecule tested, presents interesting information on the role of steric bulk. Like the flavones and honokiol, punicalagin also maintained the natively unfolded structure of αSA53T in its early stage of aggregation. Seemingly, the thin, long fibrils arising from punicalagin-treated αSA53T might not be toxic amyloid fibrils, as it exerted significant neuroprotection to αSA53T-exposed PC12 cells. These fibrils might have undergone a loss of hydrophobic surface area where ThT micelles could bind, thus diminishing fluorescence output (Khurana et al., 2005). Not surprisingly, molecular docking predicted that punicalagin was a poor binder to αSA53T, possibly due to steric hindrance as a result of its bulky size, however, it was predicted to form H-bonding interactions with key Lys 21 and 23 like other natural polyphenols tested here. Having multiple vicinal hydroxylation sites across the molecule, it is highly probable that punicalagin has several interaction modes with polar residues in an unfolded protein. Therefore, it is possible that punicalagin interacted with these predicted residues to preserve an unfolded conformation that resulted in non-amyloidogenic fibril formation. This was further supported by the TEM study following 50 h incubation. Due to the bulky size, punicalagin might have weak, non-specific interactions, not binding to a particular pocket unlike other small molecule polyphenolics. There is a correlation between the antioxidant activity of flavonoids and their *in vitro* inhibitory effect on αS fibrillization (Meng et al., 2009). We previously showed that punicalagin was capable of inhibiting amyloid β aggregation and neurotoxicity (Das et al., 2016). Given punicalagin has some similarities in terms of being an extensively hydroxylated polyphenol, its high antioxidant activity might also contribute toward its inhibitory effect.

### Novel dibenzyl imidazolidine scaffold effect on αSA53T fibrillization and neurotoxicity

Our previous study employing structure-based virtual screening for Aβ_1−42_ inhibitors identified two compounds with anti-aggregatory and neuroprotective roles *in vitro* (Das and Smid, 2017). These two synthetic compounds bearing a novel dibenzyl imidazolidine scaffold could provide insight on the effect of this scaffold on αSA53T aggregation in comparison with natural polyphenolics. TEM imaging suggests that the αSA53T fibril morphology, modified by both compounds, is different than that induced by the polyphenolics. Nonetheless, the TEM study after 50 h incubation showed that αSA53T modified by Compound 2 resembled the morphology of myricetin-incubated samples. Furthermore, there is variable effect on fibril morphology between these two compounds. In the presence of Compound 1, the small dense aggregates look different than the amorphous aggregates produced by 2-D08. The presence of both aggregates and short fibrils indicate that Compound 1 might have more than one mechanism of interaction with αSA53T during aggregation. The docking study predicted no polar interactions between Compound 1 and αSA53T and showed higher docking scores, suggesting a non-stable protein-ligand interaction. The reduction of CCSs for some αSA53T charge states in the presence of Compound 1 indicates it is less effective in stabilizing the native αSA53T unfolded state. Therefore, interaction of Compound 1 with αSA53T might not completely prevent its amyloidogenic aggregation, especially at lower concentration, unlike the natural polyphenolics and Compound 2. This was further supported by the comparative neurotoxicity observed in PC12 cells, and a higher ThT fluorescence at equimolar concentrations of protein: compound.

In the case of Compound 2, the appearance of thin, long fibrils but no dense aggregates points toward a different mechanism of interaction with αSA53T than Compound 1. There is some degree of similarity between αSA53T fibrils formed by Compound 2 and myricetin. However, Compound 2 had a mostly sterically favorable binding to αSA53T, while myricetin has both steric and polar interactions observable from the docking study. Compound 2 demonstrated significant neuroprotection and low ThT fluorescence at an equimolar concentration of protein: compound. Improved inhibition of αSA53T toxic amyloid aggregation by Compound 2 over Compound 1 is feasibly attributable to the additional -OCH3 functional group adjacent to the phenyl group in the dibenzyl imidazolidine scaffold in Compound 2. This finding is consistent with our previous study on these compounds against Aβ_1−42_, where we found that Compound 2 significantly inhibited Aβ_1−42_ aggregation in both ThT and TEM analysis, whereas Compound 1 was not able to inhibit ThT fluorescence (Das and Smid, 2017). Considering the novel aspect and effectiveness of both compounds bearing this dibenzyl imidazolidine scaffold, our findings highlight the potential of this molecular scaffold when targeted against pathological amyloidogenic proteins.

## Conclusion

Among the seven compounds tested and compared here, polyphenols such as 2-D08, honokiol, myricetin, punicalagin, and the synthetic imidazolidine, Compound 2 are the most effective inhibitors of αSA53T toxic amyloidogenic aggregation, while transilitin and other synthetic imidazolidines, such as Compound 1, were less effective. All of these compounds inhibited toxic amyloidogenic aggregation by stabilizing the native unfolded αSA53T conformations, which in turn altered αSA53T fibril morphology. This structurally diverse group of molecules could potentially facilitate improved drug design targeting the complexity of progressive neurodegenerative diseases associated with amyloidogenic protein aggregation.

## Author contributions

SD, SS, and TP conceived the presented study. SD performed the experiments, computational modeling studies and data analysis with support from TP and project supervision from SS. The manuscript was written by SD with input from all authors.

### Conflict of interest statement

The authors declare that the research was conducted in the absence of any commercial or financial relationships that could be construed as a potential conflict of interest.
