# Supplementary material for: Exploring the Structural Diversity in Inhibitors of α-Synuclein Amyloidogenic Folding, Aggregation, and Neurotoxicity
Source: Front Chem. 2018 May 25;6:181. doi: 10.3389/fchem.2018.00181 (PMC5983024; doi:10.3389/fchem.2018.00181)
Supplement: Supplementary file 1 [file Table_1.docx]

**Table 1.** Mean RFU (at plateau) and SEM values from the ThT assay at 1:2 and 1:1 (protein: compound) molar ratio.

| Test | Mean RFU at 1:2 | SEM | Mean RFU at 1:1 | SEM |
| --- | --- | --- | --- | --- |
| αSA53T alone  Compound 1  Compound 2  2-D08  Myricetin  Honokiol  Punicalagin  Transilitin | 25862  1584  453  -67.88  -1373  2183  -2168  -409.5 | 945.9  164.3  117.4  89.99  26.16  97.6  24.4  71.25 | 25083  8692  3828  2512  3164  2352  1422  4785 | 949.2  329.6  145.8  207.2  126.6  103.3  52.53  153.5 |

**Table 2.** Mean and SEM values for the cell viability assay at 1:2 and 1:1 (protein: compound) molar ratio.

| Test | Mean of % cell viability at 1:2 | SEM | Mean of % cell viability at 1:1 | SEM |
| --- | --- | --- | --- | --- |
| αSA53T alone  Compound 1  Compound 2  2-D08  Myricetin  Honokiol  Punicalagin  Transilitin | 49.16  75.62  82.97  104.6  104.6  105.1  85.86  99.17 | 4.774  0.3169  2.789  6.928  4.729  5.2  3.353  4.392 | 61.2  74.11  94.47  93.21  90.78  90.68  89.53  73.56 | 2.141  4.768  4.609  5.829  5.501  3.775  4.335  3.477 |
