# Supplementary figures and images for: Exploring the Structural Diversity in Inhibitors of α-Synuclein Amyloidogenic Folding, Aggregation, and Neurotoxicity
Source: Front Chem. 2018 May 25;6:181. doi: 10.3389/fchem.2018.00181 (PMC5983024; doi:10.3389/fchem.2018.00181)

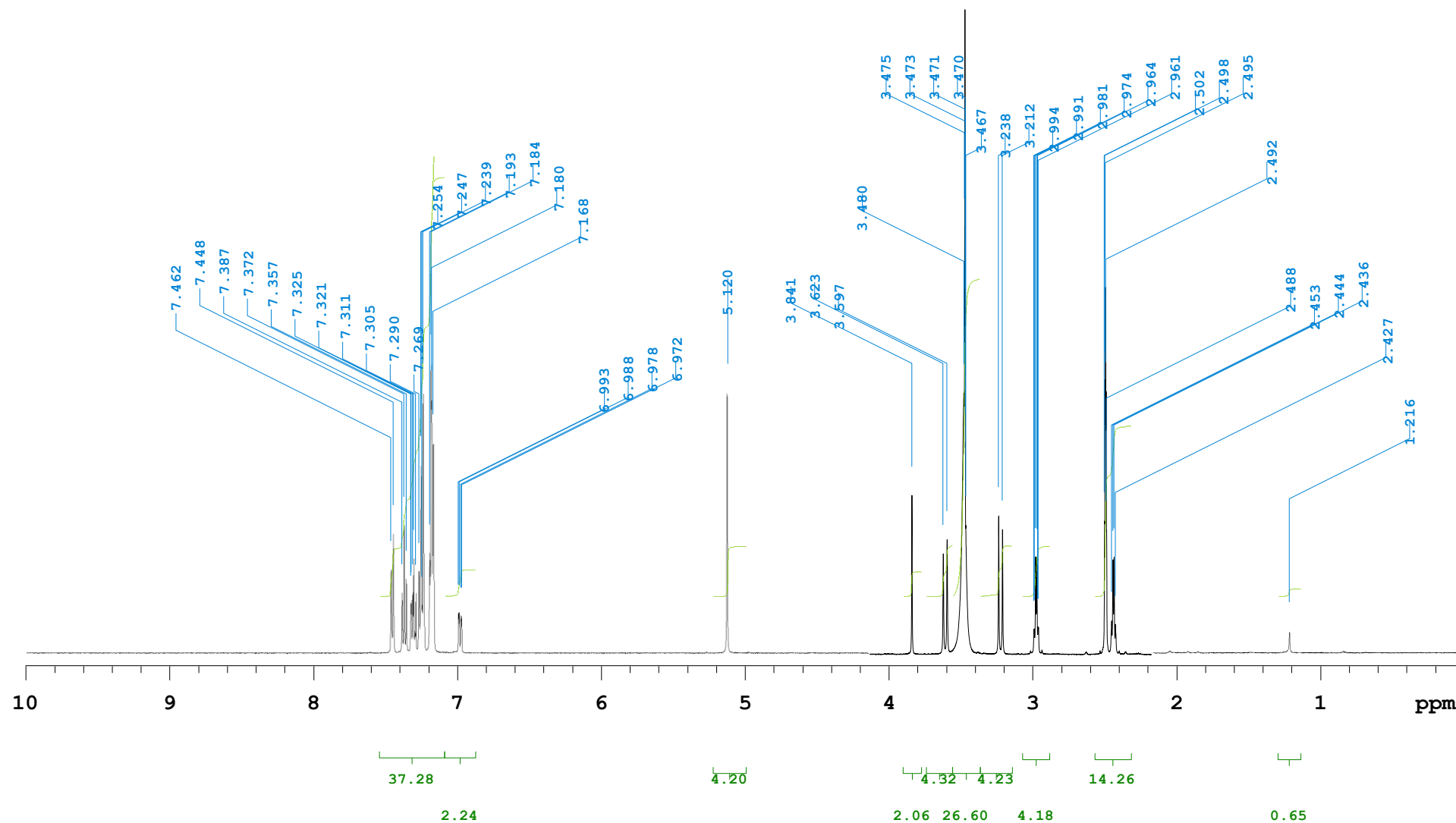

p

**Figure 1:**  $^1\text{H}$  NMR of Compound 1 (1,3-dibenzyl-2-[3-(benzyloxy)phenyl] imidazolidine).

Supplement: Supplementary file 2 [file Image_1.PDF]

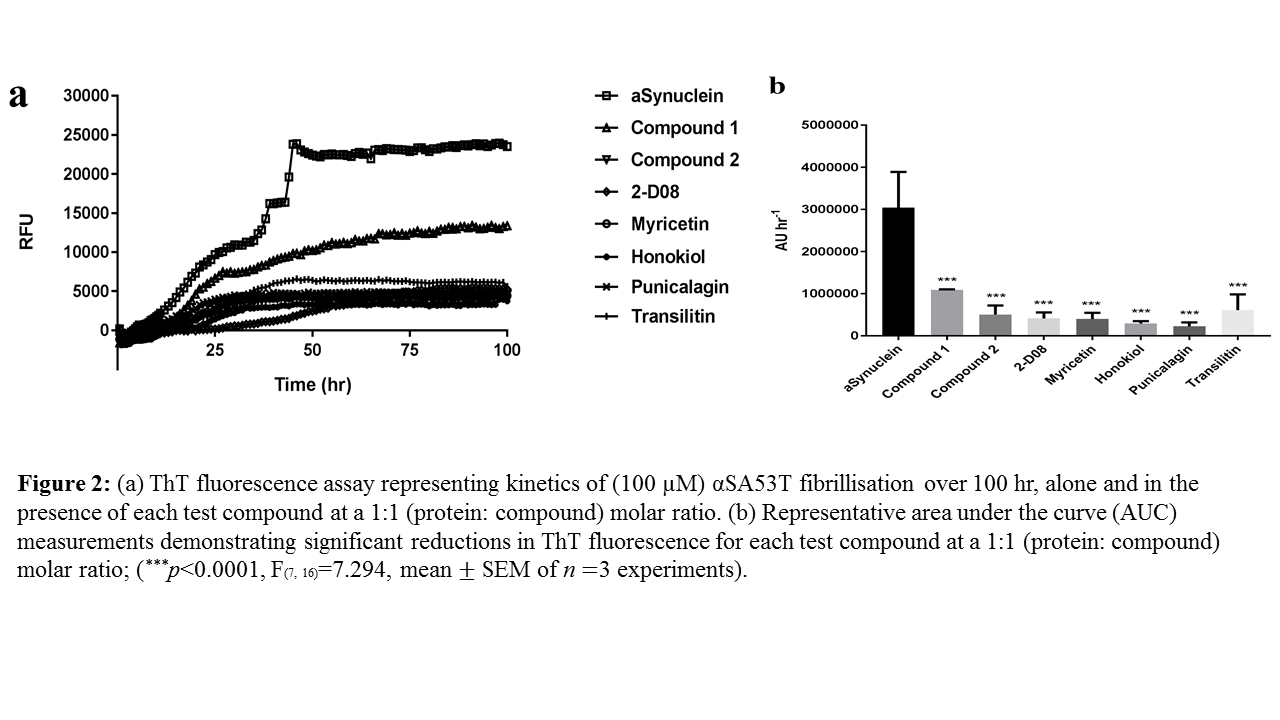

Supplement: Supplementary file 3 [file Image_2.TIF]

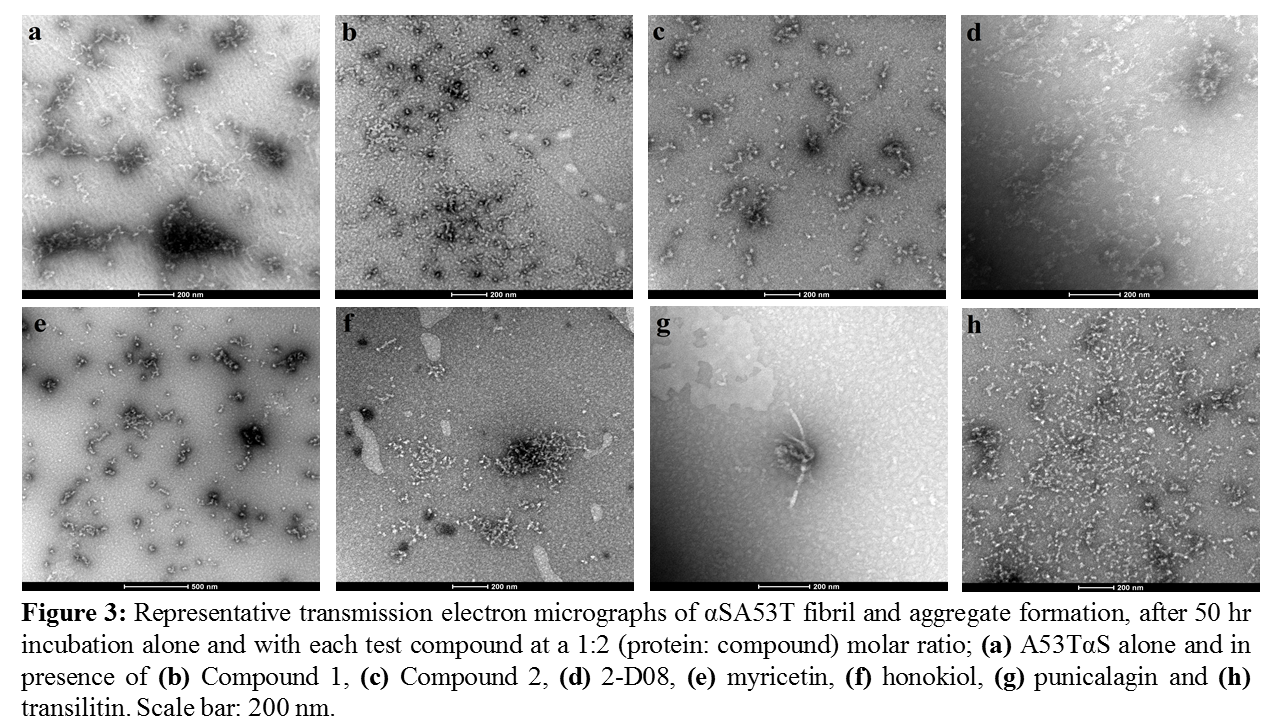

Supplement: Supplementary file 4 [file Image_3.TIF]

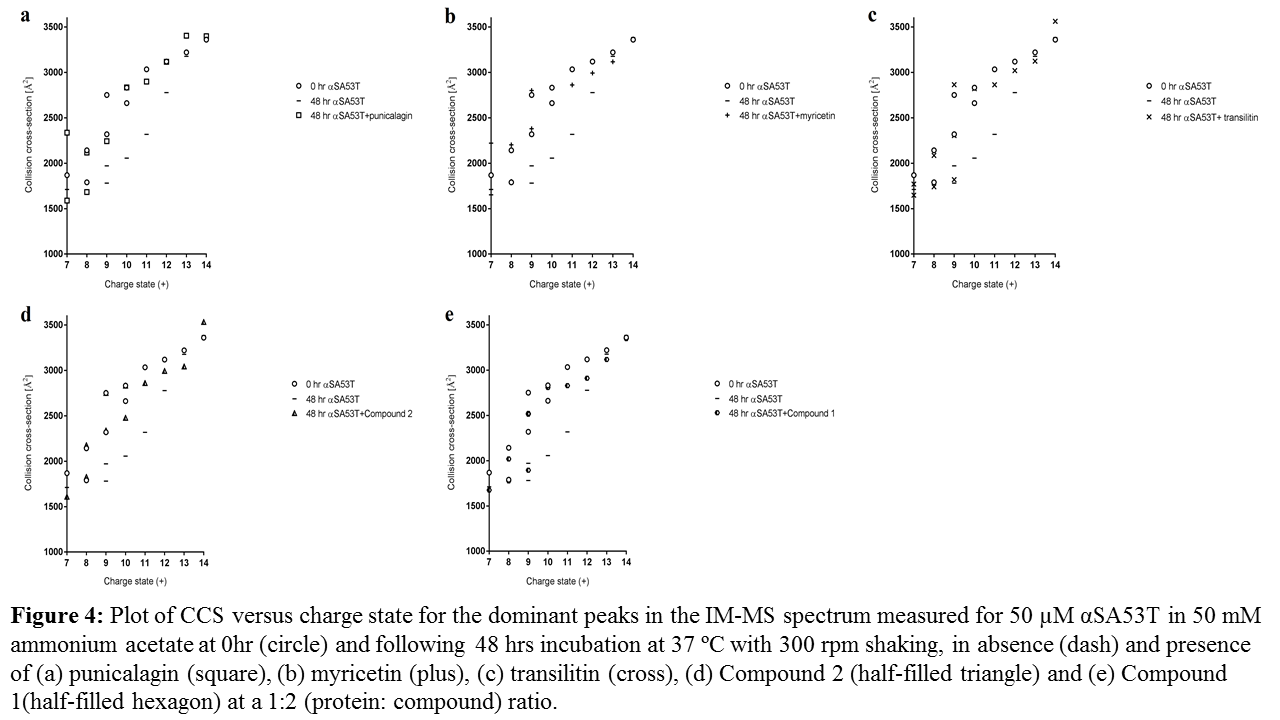

Supplement: Supplementary file 5 [file Image_4.tif]

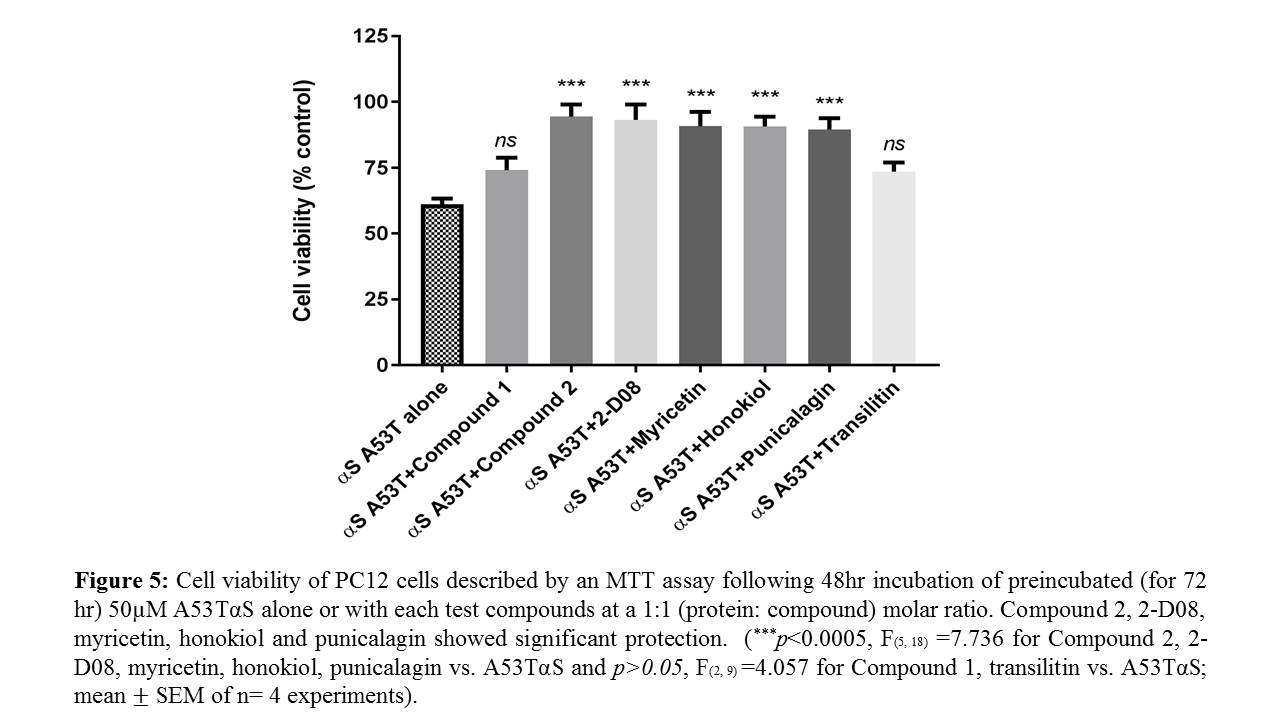

Supplement: Supplementary file 6 [file Image_5.tif]

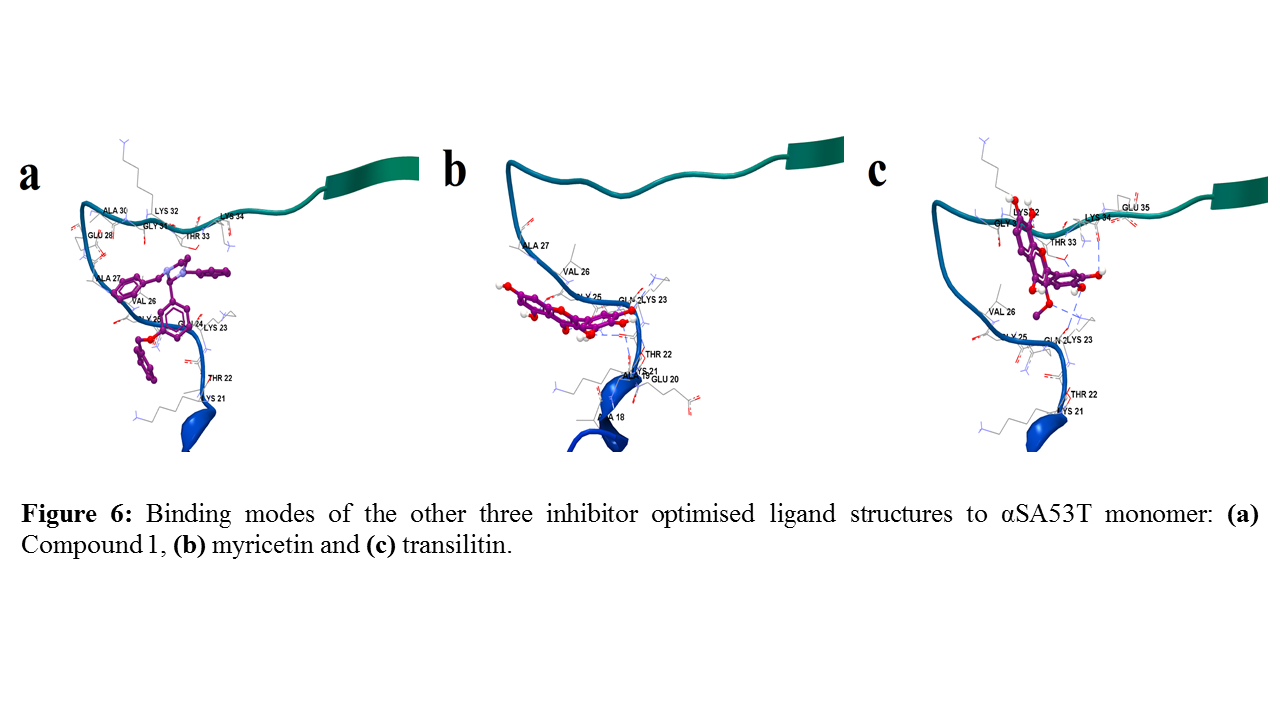

Supplement: Supplementary file 7 [file Image_6.TIF]
